# Outliers of Treatment Frequency in Retinal Vein Occlusion: 24‐Month Comparative Analysis of Fight Retinal Blindness! Practitioners

**DOI:** 10.1111/ceo.14490

**Published:** 2024-12-28

**Authors:** Theodorus Ponsioen, Yohei Hashimoto, Alessandro Invernizzi, Pierre‐Henry Gabrielle, Francisco Javier Lavid, Hemal Mehta, Rufino Silva, Nandor Jaross, David Squirrell, Louise O'Toole, Pavol Kusenda, Daniel Barthelmes, Mark Gillies, Adrian Hunt

**Affiliations:** ^1^ Department of Ophthalmology Isala Zwolle The Netherlands; ^2^ The Save Sight Institute, Sydney Medical School, the University of Sydney Sydney New South Wales Australia; ^3^ Department of Biomedical and Clinical Science Luigi Sacco Hospital, University of Milan Milan Italy; ^4^ Department of Ophthalmology Dijon University Hospital Dijon France; ^5^ Department of Ophthalmology Hospital Punta Europa Algeciras Cádiz Spain; ^6^ Department of Ophthalmology Royal Free London NHS Foundation Trust London UK; ^7^ Faculty of Medicine University of Coimbra Coimbra Portugal; ^8^ Department of Ophthalmology Coimbra Hospital and University Centre Coimbra Portugal; ^9^ Association for Innovation and Biomedical Research on Light and Image (AIBILI) Coimbra Portugal; ^10^ Australian Eye Specialists (Wyndham) Werribee Victoria Australia; ^11^ Auckland District Health Board Auckland New Zealand; ^12^ Mater Private Hospital Dublin Ireland; ^13^ Department of Ophthalmology University Hospital—St. Michael's Hospital Bratislava Slovakia; ^14^ Department of Ophthalmology University Hospital and University of Zurich Zurich Switzerland; ^15^ Department of Ophthalmology Westmead Hospital Westmead New South Wales Australia

**Keywords:** BRVO, CRVO, macula, occlusion, oedema, retinal, treatment frequency, vein

## Abstract

**Background:**

We aimed to describe a 2‐year outcome of eyes managed by practitioners benchmarked using a funnel plot by their frequency of treatment using vascular endothelial growth factor (VEGF) inhibitors for naive retinal vein occlusion (RVO).

**Methods:**

A multicentre, international, observational study of 29 doctors in 12 countries managing 1110 eyes with RVO commencing VEGF inhibitors between 1 January 2012–2022 tracked in the Fight Retinal Blindness! registry.

**Results:**

We identified 3 outlying ‘intensive’ practitioners (managing 350/1110 eyes [32%]), 22 ‘typical’ practitioners (604/1110, [54%]) and 4 outlying ‘relaxed’ practitioners (156/1110, [14%]) with respective 24‐month outcomes in Branch and Central RVO including the primary outcome, mean adjusted change in visual acuity (VA) in BRVO: +16.2, +13.6, +9.3 letters (*p* < 0.01) and CRVO: +14.2, +12.7, +4.8 letters (*p* < 0.01); adjusted change in macular thickness in BRVO −179, −150, −159 μm (*p* < 0.01) and CRVO −324, −283, −232 μm (*p* < 0.01); time‐in‐range with VA > 68 letters in BRVO 90, 78, 68 weeks (*p* < 0.01) and CRVO 69, 60, 54 weeks (*p* = 0.04); median injections 18, 13 and 10; median final injection intervals, BRVO 6, 9, 10 weeks and CRVO 6, 9 and 12 weeks; with no significant difference in adverse outcomes.

**Conclusions:**

At 24 months, the intensive practitioners were treating RVO using VEGF inhibitors with twice the frequency of the relaxed practitioners; however, their patients had gained twice (BRVO) to three times (CRVO) more letters of VA.

**Abbreviations**
BRVO: branch retinal vein occlusion
CI: confidence interval
CRVO: central retinal vein occlusion
CST: central subfield thickness
FRB!: Fight Retinal Blindness!
GAMMs: generalised additive mixed effects models
IRB: Institutional Review Board
LogMAR: logarithm of the minimum angle of resolution
MO: macular oedema
OCT: optical coherence tomography
RCT: randomised controlled trial
RWE: real‐world evidence
SD: standard deviation
VA: visual acuity
VEGF: vascular endothelial growth factor

## 1 Introduction

There is likely a range of treatment intensities amongst practitioners using vascular endothelial growth factor (VEGF) inhibitors to treat retinal vein occlusion (RVO) based on their interpretation of the evidence, regional differences in access and their individual patient's tolerance of the treatment burden.

Existing real‐world evidence has suggested a strong relationship between treatment frequency and visual acuity (VA) outcomes in RVO; however, disease severity likely contributed to that association [1]. Grouping in that evidence was by the number of injections that eyes received. The top end of injection frequency was overpopulated by eyes with lower vision and the lower end by eyes with less severe disease. This approach may have exaggerated the benefits of frequent VEGF inhibitor injections for RVO because eyes with severe disease can have very large gains in VA, whereas eyes with mild disease are limited by a ‘ceiling’ effect [2]. Quantifying the effect of treatment frequency in isolation would be better achieved if the groups were otherwise similar at baseline, each with a representative mix of patients typically encountered in routine practice.

This study used data from the Fight Retinal Blindness! (FRB!) registry to compare outcomes in patients 24 months after commencing VEGF inhibitors for treatment‐naïve RVO based on the benchmarked mean treatment frequency of their treating physician. Grouping by the treatment frequency of practitioners rather of individual eyes, along with adjustment for VEGF inhibitor utilisation and baseline difference in cohorts, aimed to minimise the confounding effect of disease severity seen in previous reports.

## 2 Methods

### 2.1 Design and Setting

This was a multicentre international observational study analysing the effect of the mean practitioner treatment frequency on 24‐month outcomes in treatment‐naïve eyes with RVO commencing VEGF inhibitors tracked in the FRB! registry. The registry is accessed free of charge via a web‐based interface or, in some centres, through automatic electronic medical record (EMR) integration [3]. The data in FRB! are of high quality, consisting of a pre‐specified minimum dataset from each visit that is only accepted when it is 100% complete and within pre‐specified ranges. There is no option for free text; all inputs are mutually exclusive, numeric or from drop‐down menus. FRB! users agree to track at least 85% of the patients they managed with the relevant condition. As the caseload of individual physicians increases, they are more likely to be managing a representative sample of the diversity of patients encountered in real‐world practice.

The FRB! registry is observational, in no way modifying the treatment decisions made by participating clinicians in consultation with their patients. Data pertaining to each visit are entered very quickly or automatically if integrated with the EMR. The baseline FRB! visit records demographic details, ocular conditions, details of pretreatment, ischaemic angiography findings and presence of glaucoma. Subsequent FRB! visits record VA in the most letters read on a logarithm of minimum angle of resolution (LogMAR) acuity chart (best of unaided, aided or pinhole), intraocular pressure, central subfield thickness (CST, in microns), the presence or absence of macular oedema (MO) judged by the treating physician, treatment given at that visit, RVO complications, procedures that day or since the last visit, adverse events and discontinuation of treatment reasons recorded when necessary with additional drop‐down menus.

Ethics and data protection were granted by the Royal Australian and New Zealand College of Ophthalmologists (HREC#16.09); Société Française d'Ophtalmologie, France (2017_CLER‐IRB_ll‐05); Mater Private Network Dublin IRB, Ireland (1/3788/2130); The Area 1 Milan Ethical Committee; Academic Medical University Centre (non‐interventional approval), Netherlands; Comissão de Ética para a Saude. Centro Hospitalar e Universitário de Coimbra, Portugal; Etická komisia UN‐Nemocnica svätého Michala, Slovakia; The Spanish IRB (Comité Etico de Investigación Medica, Hospital Clínic de Barcelona), Spain; The Cantonal Ethics Committee Zurich; and the Caldicott Guardian at the Royal Free London NHS Foundation Trust, UK. This observational study adhered to the STROBE checklists [4], the Declaration of Helsinki tenets, and ‘opt‐in’ written consent was provided in European centres and ‘opt‐out’ in Australia and New Zealand.

### 2.2 Patient Selection and Definitions

Eligible eyes were treatment‐naïve with MO due to RVO commencing treatment between 1 January 2012 and 1 January 2022 with either bevacizumab (1.25 mg Avastin; Genentech Inc., CA, USA/Roche, Basel, Switzerland), aflibercept (2 mg Eylea, Bayer) or ranibizumab (0.5 mg Lucentis, Genentech Inc/Novartis); they must have received at least three injections of ‘VEGF monotherapy’ and had at least 1 year of follow‐up (365 days) to establish treatment frequency. Eyes were excluded if they received steroids in the first year; otherwise, observations were censored after switching to steroids if it occurred in the second year. The study period was from the first treatment to the 2‐year visit closest to 730 days (2 years ± 90 days). 24‐month ‘completers’ were defined by having a 2‐year visit. The study focused on eligible practitioners (> 5 eligible eyes), meaning that some otherwise eligible eyes were excluded.

### 2.3 Outcomes

We isolated treatment frequency as the main uncontrolled variable by grouping practitioners rather than eyes, since an individual practitioner's general treatment aggression presumably remains reasonably constant irrespective of disease severity. Nevertheless, we adjusted outcomes to account for any baseline demographic differences between practitioner cohorts and for their individual practitioners' preference or limited availability to use certain VEGF inhibitors in patients that they managed.

The primary outcome was the mean adjusted change in VA from baseline to 24 months. Secondary outcomes included the mean adjusted change in CST; percentages with final VA ≥ 70 or ≤ 35 letters, and VA gain or loss ≥ 15 letters; time‐in‐range with VA > 68 letters (weeks), final interval between injections, visits and injections, 24‐month completion, laser treatments and adverse events.

### 2.4 Statistical Analysis

Clinical registries typically use funnel plots to detect poor outcomes due to atypical practice patterns of individuals that then trigger quality improvement in a learning health system through feedback [5]. Here, we used funnel plots to benchmark the average treatment frequency of individual FRB! practitioners [6]. We wanted to identify outliers with great confidence by using a 99.7% confidence interval (CI). The benefit of using a funnel plot to identify outliers, rather than standard deviation (SD) or quartiles, comes from the incorporation of statistical power through caseload; i.e., practitioners managing a large caseload would be less vulnerable to runs of severe or mild disease affecting their mean treatment frequency compared with practitioners managing only a few cases. The higher the caseload, the less likely that practitioners' treatment frequency is confounded by such bias.

Generating the funnel plot involved first plotting the mean treatment interval of eyes managed by each practitioner on the *y*‐axis against caseload on the *x*‐axis. Then, a 99.7% confidence limit (CI) at 3.0 standard errors above and below the overall mean was applied, forming the funnel that narrows with increasing caseload. This is because the standard error is the SD divided by the square root of caseload. Practitioners outside the predicted confidence limit were identified as ‘intensive’ or ‘relaxed’ outliers of treatment frequency. The mean interval between injections through 24 months for each eye was calculated after loading—either after 6 months of treatment or earlier if graded as having a resolution of MO within 6 months by the treating physician.

The adjusted VA (or CST) for BRVO and CRVO was analysed separately for each treatment frequency group from baseline to 24 months using generalised additive mixed effects models (GAMMs). The models were adjusted for which the VEGF inhibitor was initiated, baseline VA (or CST), age and nesting in bilateral cases. The outputs from these models were plotted over 24 months and provided the 24‐month adjusted outcomes including the mean adjusted change in VA (the primary outcome) and the mean adjusted change in CST. Time‐in‐range was the mean number of weeks that eyes in each group spent with VA > 68 letters, as previously described by Kozak et al. in eyes treated with VEGF therapy for diabetic macular edema [7].

Crude demographic and outcome data were summarised with counts, percentages, means, SDs or CIs, and where appropriate, medians with first and third quartiles (Q1, Q3). Statistical analysis was performed using R version 4.2.2 (http://www.R‐project.org/), utilising the *mgcv* (V1.9‐1) for GAMMs [8].

## 3 Results

### 3.1 Patient Characteristics and Disposition

We identified 1110 eyes (587 BRVO [53%], 523 CRVO [47%]) managed by 29 practitioners eligible for this analysis in 12 countries (Australia, France, Ireland, Italy, Netherlands, New Zealand, Portugal, Slovakia, South Africa, Spain, Switzerland and United Kingdom). Five hundred and seventy‐six otherwise eligible eyes were excluded from the analysis because they received ≤ 2 injections, received steroids in the first year, follow‐up was ≤ 12 months or their physician had tracked fewer than five eyes with RVO in the registry.

**FIGURE 1:**
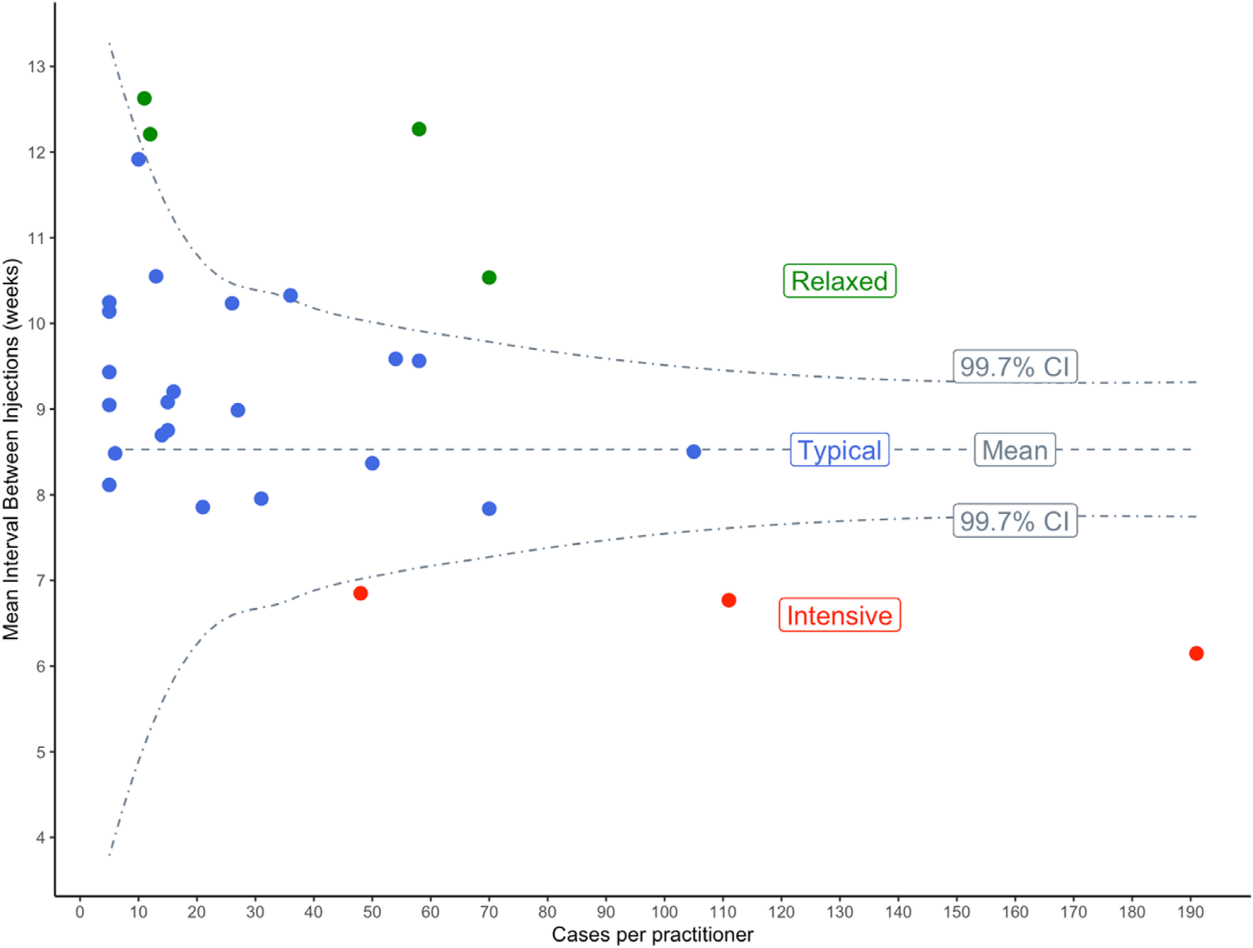
Funnel plot identifying outliers of treatment frequency in RVO based on the mean treatment interval between injections in the patients that they managed. The central dashed line is the mean treatment interval of the entire cohort. The dot‐dashed lines (the funnel) represent 3 standard errors (i.e., the 99.7% confidence interval) of deviation from the overall mean. The ‘typical’ (blue) practitioners fall within the funnel. Outlying practitioners including the ‘intensive’ (red) and ‘relaxed’ (green) practitioners fall outside the funnel.

A funnel plot identified the eligible practitioners outside the 99.7 CI as outliers of treatment frequency, including three ‘intensive’ and four ‘relaxed’ practitioners with the mean interval between injections more than 3 standard errors (99.7% CI) above or below the overall mean (Figure 1).

The ‘intensive’ outliers managed 350/1110 eyes (31%), the ‘relaxed’ outliers managed 156/1110 eyes (14%), while the remaining 22 practitioners labelled ‘typical’ managed 604/1110 eyes (54%). One practitioner was only eligible for BRVO. Table 1 describes the baseline characteristics of the eyes grouped by the treatment frequency of their practitioner. The BRVO ‘intensive’ group had higher baseline VA (*p* < 0.01) and lower baseline CST (*p* = 0.03) compared with the BRVO ‘typical’ and ‘relaxed’ groups. The characteristics of the CRVO groups were broadly similar. Bevacizumab was the commonest choice of the initial VEGF inhibitor for eyes in the ‘intensive’ group and ranibizumab for those in the ‘typical’ or ‘relaxed’ groups (*p* < 0.01, Table 1). In keeping with the routine practice, this cohort included 293/1110 eyes (26%) with baseline VA below or above the typical inclusion criteria of major trials (19–73 letters) [2, 9, 10, 11, 12, 13, 14].

**TABLE 1:** Baseline demographics: BRVO and CRVO eyes grouped by the treatment frequency of the practitioner managing their care.

	BRVO (587 eyes)			*p* ^**a**^	CRVO (523 eyes)			*p* ^**a**^
	Intensive	Typical	Relaxed		Intensive	Typical	Relaxed	
Practitioners, *n*	3	22	4		3	21	4	
Eyes, *n*	161	337	89		189	267	67	
Patients, *n*	159	332	87		187	261	66	
Mean interval, weeks (SD)	6.3 (2)	9.1 (3.3)	11.5 (4)	**< 0.01**	6.6 (2.7)	8.7 (3.3)	11.5 (4.6)	**< 0.01**
VA, letters (SD)	62.2 (17)	57.2 (17.3)	57.8 (17.7)	**< 0.01**	41.7 (26.5)	44.4 (25.4)	47.1 (21.4)	0.27
≤ 35 letters, %	7%	13%	16%	0.10	39%	36%	30%	0.44
≥ 70 letters, %	42%	28%	37%	**< 0.01**	16%	16%	16%	0.99
CST, μm (SD)	446 (134)	485 (171)	453 (161)	**0.03**	610 (246)	614 (237)	610 (220)	0.91
Age, years (SD)	70 (12)	71 (11)	72 (10)	0.12	71 (12)	72 (12)	70 (11)	0.65
Gender, % female	55%	56%	55%	0.98	43%	42%	45%	0.92
Initial injection				**< 0.01** ^**b**^				**< 0.01** ^**b**^
Bevacizumab, %	116 (72%)	90 (27%)	6 (7%)		93 (49%)	43 (16%)	7 (10%)	
Ranibizumab, %	23 (14%)	128 (38%)	49 (55%)		52 (28%)	111 (42%)	33 (49%)	
Aflibercept, %	22 (14%)	119 (35%)	34 (38%)		44 (23%)	113 (42%)	27 (40%)	

*Note: P* values < 0.05 (bold) were considered significant.

Abbreviations: BRVO = branch retinal vein occlusion, CRVO = central retinal vein occlusion, CST = central subfield thickness, *n* = number, SD = standard deviation, VA = visual acuity.

^a^ *p*‐values were otherwise derived using analysis of variance (ANOVA).

^b^ Chi‐square test on 3 × 3 contingency tables.

### 3.2 Visual and Anatomical Outcomes

**FIGURE 2:**
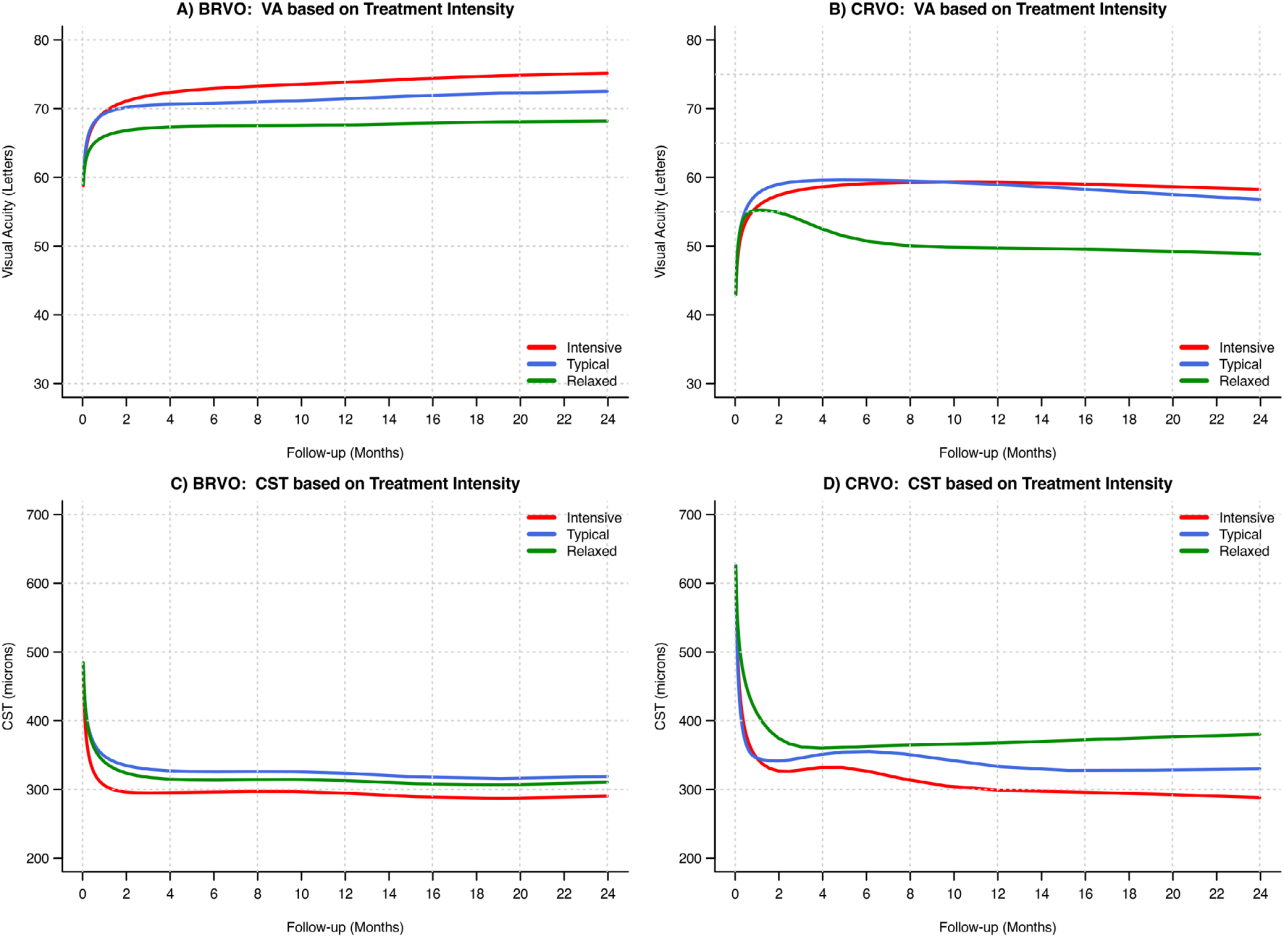
Adjusted mean visual acuity (VA) and central subfield thickness (CST) through 24 months by the RVO type (branch: BRVO or central: CRVO) and grouped by treatment frequency of the practitioner managing their care. Adjustment was performed using generalised additive mixed effects models (GAMMs) accounting for difference in the initial VEGF inhibitor, baseline VA (or CST), age and nesting in bilateral cases.

The primary outcome of the adjusted mean change in VA for ‘intensive’, ‘typical’ and ‘relaxed’ groups with BRVO was +16.2, +13.6, and +9.3 letters (*p* < 0.01) and with CRVO, it was +14.2, +12.7, and +4.8 (*p* < 0.01), respectively (Table 2, Figure 2). The adjusted mean change in CST for ‘intensive’, ‘typical’ and ‘relaxed’ groups with BRVO was −179, −150, and −159 μm (*p* < 0.01), respectively; with CRVO, it was −324, −283, and −232 μm (*p* < 0.01), respectively. Adjustment of outcomes was applied to address the significant heterogeneity of utilisation of different VEGF inhibitors and for significant difference in baseline characteristics, particularly in the BRVO groups. Adjusted VA and adjusted CST are plotted in Figure 2. The unadjusted outcomes are also described in Table 2. In the subset of eyes in our cohort with baseline VA within the inclusion criteria of major RVO trials (19–73 letters), the adjusted mean change in VA for ‘intensive’, ‘typical’ and ‘relaxed’ groups with CRVO was +12.0, +9.1, and +0.6 letters (*p* < 0.01), and for BRVO, it was +17.7, +13.9, and +10.3 letters (*p* < 0.01), respectively.

**TABLE 2:** 24‐month outcomes: BRVO and CRVO eyes grouped by the treatment frequency of the practitioner managing their care.

	BRVO (587 eyes)			*p*	CRVO (523 eyes)			*p*
	Intensive	Typical	Relaxed		Intensive	Typical	Relaxed	
Eyes, *n*	161	337	89		189	267	67	
Completers, *n* (%)	133 (83%)	271 (80%)	65 (73%)	0.18	154 (81%)	202 (76%)	52 (78%)	0.34
Initial VA, mean (SD), letters	62.2 (17)	57.2 (17.3)	57.8 (17.7)	**< 0.01**	41.7 (26.5)	44.4 (25.4)	47.1 (21.4)	0.27
Final VA, mean (SD), letters	76.6 (14.5)	70.2 (16.1)	66.9 (17.4)	**< 0.01**	57.6 (27.4)	56.8 (25.3)	50.3 (24)	0.13
≤ 35 letters, %	3%	4%	10%	0.05	22%	19%	22%	0.78
≥ 70 letters, %	80%	68%	62%	**< 0.01**	46%	38%	27%	**0.02**
Gain ≥ 15 letters, %	48%	40%	35%	0.10	51%	45%	36%	0.10
Loss ≥ 15 letters, %	1%	4%	9%	**0.01**	8%	12%	22%	**0.01**
ΔVA, mean (95% CI), letters	+14 (12, 17)	+13 (11, 15)	+9 (5, 13)	0.06	16 (12, 19)	12 (9, 16)	3 (−3, 9)	**< 0.01**
*Adjusted* Δ VA mean (95% CI), letters^a^	+16.2 (14.3, 18.2)	+13.6 (12.0, 15.2)	+9.3 (6.6, 12.0)	**< 0.01**	+14.2 (10.4, 18.0)	12.7 (9.4, 16.0)	4.8 (−0.6, 10.3)	**< 0.01**
Final CST, mean (SD), μm	278 (56)	307 (96)	300 (142)	**< 0.01**	292 (123)	335 (171)	361 (188)	**< 0.01**
Δ CST, mean (95% CI), μm	−168 (−191, −145)	−177 (−197, −157)	−154 (−196, −111)	0.54	−319 (−357, −281)	−280 (−312, −247)	−248 (−314, −183)	0.15
*Adjusted* Δ CST, mean (95% CI), μm^a^	−179 (−166, −193)	−150 (−138, −163)	−159 (−142, −176)	**< 0.01**	−324 (−299, −349)	−283 (−263, −302)	−232 (−199, −265)	**< 0.01**
Injections, median (Q1, Q3)^b^	18 (14, 23)	12 (9, 16)	10 (7, 12)	—	18 (12, 23)	14 (11, 17)	10 (7, 12)	—
Last interval, median (Q1, Q3) weeks^b^	6 (4, 10)	9 (6, 13)	10 (7, 17)	—	6 (4, 10)	9 (7, 12)	12 (9, 17)	—
Visits, median (Q1, Q3)^b^	22 (17, 26)	16 (13, 20)	18 (15, 21)	—	22 (18, 26)	19 (16, 23)	18 (16, 22)	—
Visits with injections, %	82%	72%	57%	**< 0.01**	75%	70%	57%	**< 0.01**
Time‐in‐range (TIR)^c^								
Achieved VA > 68 letters, %	88%	89%	89%	0.96	68%	74%	63%	0.16
TIR, weeks, mean (% weeks)	90 (87%)	78 (76%)	68 (67%)	**< 0.01**	69 (67%)	60 (58%)	54 (51%)	**0.04**
Switched VEGF inhibitor, *n* (%)	34 (21%)	77 (23%)	11 (12%)	**< 0.01**	41 (22%)	44 (16%)	7 (10%)	**< 0.01**

*Note: P* values < 0.05 (bold) were considered significant.

Abbreviations: BRVO = branch retinal vein occlusion, CI = confidence interval, CRVO = central retinal vein occlusion, CST = central subfield thickness, *n* = number, Q1 = first quartile, Q3 = third quartile, SD = standard deviation, TIR = time‐in‐range (VA > 68 letters), VA = visual acuity, VEGF = vascular endothelial growth factor.

^a^ Adjusted for baseline VA, CST, age, initial VEGF inhibitor and nesting in bilateral cases using generalised additive mixed effects models.

^b^ Calculated in 24‐month completers, but *p*‐values were not calculated as the groups were formed based on the difference in these parameters.

^c^ Includes completers that achieved > 68 letters within the study duration, mean time expressed in weeks and as a mean percentage of the 104‐week study. The p‐values were derived using the analysis of variance (ANOVA) for continuous variables.

### 3.3 Time‐in‐Range Analysis

The proportion of eyes achieving VA > 68 letters at any point during the study was 89% in BRVO and 71% in CRVO, which was independent of treatment frequency (*p* = 0.96, *p* = 0.16, Table 2). In the subset of eyes that did achieve VA > 68 letters, it was the mean time‐in‐range that differed significantly between ‘intensive’, ‘typical’ or ‘relaxed’ groups for both BRVO (90, 78, 68 weeks [*p* < 0.01]) and CRVO (69, 60, 54 weeks [*p* = 0.04]).

### 3.4 Injections, Visits and Treatments

In keeping with the study design, more frequent treatment groups had more injections, along with more visits and shorter final intervals between injections at 24 months (Table 2). This equated to around 80% more injections by ‘intensive’ practitioners compared with that of ‘relaxed’ practitioners. The completers in the ‘intensive’, ‘typical’ and ‘relaxed’ groups with BRVO received a median of 18, 12 and 10 injections, at 22, 16 and 18 visits and a median final treatment interval of 6, 9 and 10 weeks, respectively; the eyes with CRVO received a median of 18, 14 and 10 injections, at 22, 19 and 18 visits and a median final treatment interval of 6, 9 and 12 weeks at 24 months, respectively. In the second year of treatment, the median injections delivered in the ‘intensive’, ‘typical’ and ‘relaxed’ groups were in BRVO 7, 4 and 4 and in CRVO 8, 6 and 4, respectively.

Switching between VEGF inhibitors occurred in 214/1110 eyes (19%) at a median (Q1, Q3) of 276 days (169, 445). Switching occurred least in the relaxed groups (*p* < 0.01, Table 2). Laser treatment was delivered more frequently by the ‘intensive’ practitioners compared with that of the ‘typical’ or ‘relaxed’ practitioners including focal laser in BRVO (27%, 5%, 1% [*p* < 0.01]), sectoral laser in BRVO (20%, 16%, 12% [*p* = 0.30]) and PRP laser in CRVO (41%, 21%, 24% [*p* < 0.01]). Cataract surgery was performed more often in the BRVO ‘relaxed’ group compared with the ‘typical’ or ‘intensive’ BRVO groups (24%, 7%, 5% [*p* < 0.01]) but it was not significantly different in CRVO (17%, 8%, 10% [*p* = 0.13]).

### 3.5 Non‐Completion

The 24‐month completion rate was 79% overall (877/1110), similar amongst the groups, but non‐completers had less impressive outcomes. The outcomes were imputed in eyes that did not complete the study within the models. Nevertheless, at the final review, the 118/587 (20%) BRVO non‐completers had gained a mean of +9 letters compared with +14 letters in BRVO completers (*p* < 0.01). The 115/523 (22%) CRVO non‐completers gained +8 letters compared with +14 letters in CRVO completers (408/523 [78%]; *p* = 0.05). Reasons for non‐completion were documented in 152/233 (65%) of non‐completers: most were likely unrelated to poor outcomes, including (part of the 65%), ‘going to another doctor’ (16%), ‘treatment success’ (13%) and ‘deceased’ (24%), while some were likely related to a poor outcome including ‘futility of treatment’ (5%), ‘medical contraindication’ (1%) and ‘patient declined treatment’ (5%).

### 3.6 Maximum and Minimum Treatment Frequencies

The single most intensive practitioner in our study achieved better visual outcomes than other intensive practitioners. This prompted a post hoc analysis, of the 350/1110 eyes (32%) in the intensive group, that compared the single most intensive practitioner with the two other intensive practitioners (Tables 3 and 4). The most treatment‐intensive practitioner commenced treatment with bevacizumab exclusively, in 191 eyes with significantly higher baseline VA (BRVO; *p* = 0.03, CRVO; *p* = 0.03) than other intensive practitioners treating 159 eyes. The most treatment‐intensive practitioner delivered higher median injections compared with other intensive practitioners in both BRVO and CRVO over the 2‐year study (20 vs. 16; *p* < 0.01, 21 vs. 15; *p* < 0.01). In the second year, the most treatment‐intensive practitioner delivered a median of eight injections in BRVO and nine in CRVO. The single most intensive practitioner treated 39% of BRVO eyes under their care with focal laser, whereas other intensive practitioners treated only 5% of BRVO eyes. The mean final VA in eyes treated by the most treatment‐intensive practitioner was higher in BRVO (79 vs. 72 letters; *p* < 0.01) and CRVO (66 vs. 51 letters; *p* < 0.01), including significantly fewer eyes with final VA ≤ 35 letters (BRVO *p* = 0.05, CRVO *p* = 0.01) compared with other intensively treated eyes. The time‐in‐range (weeks, VA > 68 letters) was higher in CRVO eyes treated by the most intensive practitioner than that in other intensive CRVO eyes (74% vs. 63%; *p* = 0.04). The 24‐month anatomic outcomes were similar between members of the intensive group with CRVO or BRVO. We analysed the outcomes of relaxed practitioners in a similar way but found outcomes similar amongst the relaxed practitioners.

**TABLE 3:** Baseline demographics of BRVO and CRVO eyes treated by the most intensive practitioner compared with the two other intensive practitioners.

	Intensive BRVO (156 eyes)		*p* ^**a**^	Intensive CRVO (189 eyes)		*p* ^**a**^
	Most intensive	Other intensive		Most intensive	Other intensive	
Practitioners, *n*	1	2		1	2	
Eyes, *n*	106	55		85	104	
Patients, *n*	105	54		85	102	
Mean interval, weeks (SD)	6.0 (1.9)	6.7 (2.1)	0.05	6.3 (3)	6.9 (2.5)	0.56
VA, letters (SD)	64.3 (15)	58.2 (19.9)	**0.03**	46 (24.1)	38.2 (27.9)	**0.04**
≤ 35 letters, %	4%	15%	**0.02**	28%	47%	**0.02**
≥ 70 letters, %	45%	35%	0.24	13%	18%	0.24
CST, μm (SD)	444 (131)	449 (142)	0.85	602 (200)	619 (283)	0.85
Age, years (SD)	69.6 (12)	69.9 (12.7)	0.86	69.6 (12.4)	71.6 (11.8)	0.86
Gender, % female	58%	51%	0.50	39%	47%	0.50
Initial injection						
Bevacizumab, %	100%	18%	**< 0.01** ^**b**^	100%	8%	**< 0.01** ^**b**^
Ranibizumab, %	—	42%		—	50%	
Aflibercept, %	—	40%		—	42%	

*Note: P* values < 0.05 (bold) were considered significant.

Abbreviations: BRVO = branch retinal vein occlusion, CRVO = central retinal vein occlusion, CST = central subfield thickness, n = number, SD = standard deviation, VA = visual acuity.

^a^ *p*‐values were derived using analysis of variance (ANOVA).

^b^ Chi‐square test on 3 × 3 contingency tables.

**TABLE 4:** Outcomes in BRVO and CRVO eyes treated by the most treatment‐intensive practitioner compared with the two other intensive practitioners at 24 months.

	Intensive BRVO (156 eyes)		*p*	Intensive CRVO (189 eyes)		*p*
	Most intensive	Other intensive		Most intensive	Other intensive	
Eyes, *n*	106	55		85	104	
24 m completers, *n* (%)	92 (87%)	41 (75%)	0.08	79 (93%)	75 (72%)	**<0.01**
24 m VA, mean (SD), letters	79 (11)	72 (19)	**< 0.01**	66 (22)	51 (29)	**< 0.01**
≤ 35 letters, *n* (%)	1%	7%	0.05	13%	29%	**0.01**
≥ 70 letters, *n* (%)	83%	75%	0.21	52%	41%	0.19
Gain ≥ 15 letters, *n* (%)	47%	49%	0.87	61%	42%	**0.01**
Loss ≥ 15 letters, *n* (%)	1%	2%	1.00	5%	11%	0.18
Δ VA, mean (95% CI), letters	15 (12, 17)	14 (10, 18)	0.68	20 (15, 24)	13 (8, 18)	0.05
24 m CST, mean (SD), μm	275 (47)	282 (72)	0.64	280 (69)	303 (158)	0.89
Δ CST, mean (95% CI), μm	−169 (−196, −142)	−166 (−211, −121)	0.93	−322 (−368, −275)	−316 (−374, −258)	0.90
Injections, median (Q1, Q3)^a^	20 (15, 25)	16 (12, 19)	**< 0.01**	21 (14, 25)	15 (12, 19)	**< 0.01**
Final interval, median (Q1, Q3) weeks^a^	6 (4, 10)	8 (6, 9)	0.26	6 (4, 10)	6 (4, 10)	0.67
Visits, median (Q1, Q3)^a^	22 (17, 27)	21 (18, 25)	0.68	22 (17, 26)	22 (19, 27)	0.16
Visits with injections, %	88%	70%	**< 0.01**	87%	66%	**< 0.01**
Time‐in‐range (TIR)^b^						
Achieved VA > 68 letters, %	91%	80%	0.13	71%	65%	0.36
TIR, weeks, mean (% weeks)	90 (86%)	90 (88%)	0.97	74 (72%)	63 (60%)	**0.04**
Switched VEGF inhibitor, *n* (%)	17 (16%)	17 (31%)	**0.04**	15 (18%)	26 (25%)	0.29

*Note: P* values < 0.05 (bold) were considered significant.

Abbreviations: CI = confidence interval, CST = central subfield thickness, *n* = number, Q1 = first quartile, Q3 = third quartile, SD = standard deviation, VA = visual acuity, VEGF = vascular endothelial growth factor.

^a^ Calculated in 24‐month completers.

^b^ Includes completers that achieved > 68 letters within the study duration, mean time expressed in weeks and as a mean percentage of the 104‐week study.

### 3.7 Adverse Events

Neovascular complications in either the anterior or posterior segment occurred in 58/1110 eyes (5%). The neovascular complications occurred at similar rates in the ‘intensive’, ‘typical’ and ‘relaxed’ groups with BRVO (1%, 3%, 2% [*p* = 0.55]) and CRVO (6%, 10%, 9% [*p* = 0.27]), though eyes managed by the most treatment‐intensive practitioner had very low rates (BRVO 0%, CRVO 2%). There were 11 eyes with CRVO that developed rubeosis unrelated to treatment frequency (*p* = 0.77), and the mean final VA was seven letters; all were treated with PRP laser. New macular changes including epiretinal membrane, macular hole, pigment change or atrophy occurred during the study at similar or sporadic rates in the ‘intensive’, ‘typical’ and ‘relaxed’ groups with BRVO (10%, 10%, 8%; *p* = 0.84) and CRVO (19%, 11%, 20%; *p* = 0.02). The mean final VA in eyes with new macular changes was 60 letters in BRVO and 40 letters in CRVO. Of 14 518 injections delivered in the study, there were four cases of infectious endophthalmitis (rate: 0.03%, one ‘intensive’, three ‘typical’), three cases of non‐infectious endophthalmitis (rate: 0.02%, one ‘intensive’, one ‘typical’), one retinal detachment (rate: 0.01%, ‘typical’), but no recorded cases of intraocular inflammation or vasculitis.

## 4 Discussion

We found the funnel plot helpful in categorising the relative treatment frequency of practitioners commencing VEGF inhibitors for treatment‐naïve RVO in routine care over 24 months. Patients who saw practitioners who on average performed more injections gained more letters compared to those practitioners who treated less. One highly intensive practitioner clearly had superior results to any other. On the one hand, these findings suggest that the funnel plot can be used to identify and notify relaxed practitioners of their status benchmarked against their peers. On the other, the superior outcomes of the intensive practitioners indicate what is achievable with the more frequent VEGF inhibitor therapy for RVO routine care.

The primary outcome of the adjusted 24‐month mean change in VA from baseline was significantly greater with more frequent treatment of both CRVO (‘intensive’: +14.2, ‘typical’: +12.7, ‘relaxed’: +4.8 letters; *p* < 0.01) and BRVO (+16.2, +13.6, +9.3 letters; *p* < 0.01). The mean adjusted 24‐month change in CST was significantly greater with more frequent treatment of both CRVO (−324, −283, −232 μm; *p* < 0.01) and BRVO (−179, −150, −159 μm; *p* < 0.01).

As anticipated from the study design, the frequency of injections was higher in eyes treated by ‘intensive’ practitioners compared with that in ‘relaxed’ practitioners (median injections, 18 vs. 10). At 24 months, the ‘intensive’ groups were being treated with nearly twice the frequency of eyes in the ‘relaxed’ groups (final mean treatment interval, 6 weeks vs. 10–12 weeks). Visits were only modestly more frequent by group over the entire 24 months (median, 22 in ‘intensive’ vs. 18 in ‘relaxed’ groups). It was the higher proportion of visits at which treatment was given in the intensive group (75%–80% visits), compared with that in the relaxed group (57% visits), which really lifted the overall treatment frequency. One could argue that burden is not only measured by injection numbers but also by the number of times patients and their caregivers have to attend for review.

Eyes with very poor VA can often have very large improvements in VA [15]. Most RVO trials required baseline VA between 19 and 73 letters for inclusion [2, 9, 10, 11, 12, 13, 14]. Considering only the subset of our cohort with ‘trial‐eligible’ baseline VA, we found that this subset when treated with an ‘intensive’ regimen achieved gains (CRVO: +12.0 letters, BRVO: +17.7 letters) comparable to those of the RCTs (CRVO: +10 to +16 letters, BRVO: +15 to +18 letters) [13, 16, 17, 18, 19, 20, 21], whereas ‘typical’ and ‘relaxed’ regimens were associated with less impressive outcomes more typical of evidence derived from routine care [14, 19, 22, 23, 24, 25].

The trend for better outcomes with increasing treatment frequency extended up to the single most intensive practitioner. The most intensive practitioner managed 191 eyes with RVO, maintaining high rates of a 2‐year completion (87%–93%). We were surprised by the exceptionally large gains in VA observed in these eyes, particularly as they had higher baseline VA, along with greater likelihood of a ceiling effect limiting outcomes, when compared with other intensively treated (CRVO: 46 vs. 38 letters, BRVO: 64 vs. 58 letters). Eyes treated by this most intensive practitioner achieved a mean change in VA of +20 letters from baseline in CRVO by treating at 88% of 22 visits, whereas the other intensive practitioners reviewed their patients as frequently but treated with more likely a pro‐re‐nata approach (70% of 21 visits involved treatment) to achieve the mean change in VA of +13 letters. The most intensive practitioner applied a similar frequency of treatment in eyes with BRVO, matching the mean change in VA (14–15 letters) of other intensive practitioners despite the higher baseline VA.

A trait of the ‘intensive’ outliers was the maintenance of frequent treatment through the second year of the 24‐month study. Trial evidence has demonstrated how a more relaxed regimen in the second year can erode outcomes. The SCORE2 study reported mean changes in VA at 12 months in CRVO (including Hemi‐RVO) with aflibercept or bevacizumab of +21.6 and +21.7 letters, but those early gains had eroded to +14.2 letters at 24 months, with reduced treatment frequency in the second year (3.6–4.6 injections, from 12 to 24 months)—a frequency of treatment that was similar to our ‘relaxed’ group (median four injections, from 12 to 24 months) [15].

In our study, the ‘relaxed’ outliers had particularly poor control of CST in CRVO for 2 years and poorer visual outcomes compared with the ‘typical’ and ‘intensive’ groups. The detrimental effect of the ‘relaxed’ treatment of BRVO on outcomes seemed less marked than it was in CRVO in our study. This was in keeping with the HORIZON open label extension study (second year of treatment) where participants that had already completed the BRAVO and CRUISE studies were allowed to have reviews extended out to 3 months [14, 16, 19, 26, 27]. There was a generalised loss of vision during that second year, particularly for eyes with CRVO, with few injections being delivered (CRVO: 2.0–3.8 injections, BRVO: 2.0–2.4 injections). The authors concluded that patients with RVO, particularly those with CRVO, were prone a vision loss when reviewed and treated less frequently.

The observation in our study that a similar proportion of eyes achieved VA > 68 letters (approx. 20/40) at some point suggests similar potential for good outcomes across all the groups. How long eyes stayed at that level was what differentiated the groups. Intensively treated eyes had significantly longer ‘time‐in‐range’ with VA > 68 letters (BRVO: *p* < 0.01, CRVO: *p* = 0.04). The longer time‐in‐range for the BRVO ‘intensive’ group comes with one caveat: higher baseline VA (*p* < 0.01) compared with the BRVO ‘typical’ and ‘relaxed’ groups. In CRVO particularly, perhaps the current degree of extending treatment intervals in the routine clinical practice results in degradation of the initial VA gains.

The present study graded the treatment frequency of practitioners rather than individual eyes to avoid the potential confounding effect of disease severity. We wanted to maintain the patient samples presenting to each practitioner rather than split them up. We also adjusted for baseline differences and VEGF inhibitor utilisation. The Vestrum Health Database reported a similar range of outcomes based on treatment frequency in RVO (+2.0 to +15.5 letters in CRVO and +2.9 to +13.1 letters in BRVO), but in that study, the eyes treated with increasing frequency had progressively lower baseline VA [1]. The range of gains in VA in the present study adds to the existing evidence; the methodology also likely isolates the effect on outcomes of treatment frequency from disease severity.

This study has some limitations common to observational analyses. We report and discuss the relative benefits of different levels of treatment frequency while not having the power to demonstrate any significant association with the risk of endophthalmitis, though it makes sense to assume the risk would likely be proportional to injection frequency. The range of outcomes using currently available VEGF inhibitors reported here can at least inform the management of patients in keeping with their individual tolerance of risk and treatment burden. Our conclusions are based on the difference in outcomes in eyes treated by only three intensive and four relaxed outliers, compared with 22 typical practitioners; however, the proportion of patients treated by those outliers was quite large. We adjusted for the effect of which the VEGF inhibitor was initiated; however, we did not account for any effect caused by switching VEGF inhibitors. The switching rates were below 20%, similar to other FRB! RVO cohorts, and occurred between the first‐generation VEGF inhibitors with which only small differences in visual outcomes have been demonstrated in CRVO only [2, 28]. Neither neovascularisation (combined anterior or posterior) nor rubeosis was found to be significantly associated with injection frequency, though the most intensive practitioner had very low rates of these. We cannot be sure that users fulfilled their agreement to track at least 85% of the patients they managed with the relevant condition. We report some anomalous outcomes such as the rates of macular changes affecting vision during the study in CRVO occurring in around 20% of eyes in ‘intensive’ and ‘relaxed’ groups but in only 10% in the ‘typical’ frequency CRVO group: the higher rates of cataract surgery in the ‘relaxed’ group and higher rates of laser in the ‘intensive’ group. We assume that cataract surgery may be more common in physicians with a more general focus. One would think that more frequent cataract surgery may have improved outcomes in the relaxed group. It was interesting that the single most intensive practitioner was responsible for the higher rate of focal laser in the intensive group. There is limited evidence to suggest that focal laser in BRVO can have an adjunctive benefit to visual outcomes or reduce the number of injections required in eyes already receiving VEGF inhibitors [29, 30, 31]. Because of anonymisation of data, we can offer no further insight into how the most intensive practitioner achieved such high completion rates despite what appeared to be a very high burden of therapy. Perhaps, newer agents will offer greater durability to offset the sub‐optimal levels of disease control seen anatomically in many of the CRVO eyes treated with either ‘typical’ or ‘relaxed’ intensity while also reducing the sheer number of injections required to achieve optimal outcomes seen in the ‘intensive’ groups with either BRVO or CRVO in our study [32].

We found a strong association between treatment frequency and outcomes of VEGF inhibition in RVO at 24 months by benchmarking practitioners with a funnel plot. Our findings suggest that very frequent treatment in RVO can achieve outcomes matching VEGF trial results in the real‐world setting while also highlighting the pervasiveness of undertreatment, even perhaps in doctors with ‘typical’ levels of treatment intensity. When selecting a treatment regimen, it should balance the individual expectations of patients in keeping with their tolerance of risk and treatment burden.

## Conflicts of Interest

M.G. and D.B. are the inventors of the software used to collect the data for this analysis. The following authors are members of the advisory boards of Novartis and Bayer (P.‐H.G., L.O.T., T.P. and M.G.), Roche (A.H., M.G. and T.P.), Allergan (P.‐H.G. and M.G.), and Horus and Zeiss (P.‐H.G.). Honoraria were reported from Bayer and Novartis (P.‐H.G. and L.O.T.); travel expenses were from Novartis, Bayer and Roche (L.O.T. and A.H.); D.B. received a research grant from Novartis.

## Data Availability

Research data are not shared.
